# Hepatic ILC2 activity is regulated by liver inflammation-induced cytokines and effector CD4^+^ T cells

**DOI:** 10.1038/s41598-020-57985-w

**Published:** 2020-01-23

**Authors:** Silja Steinmann, Marek Schoedsack, Fabian Heinrich, Philippe C. Breda, Aaron Ochel, Gisa Tiegs, Katrin Neumann

**Affiliations:** Institute of Experimental Immunology and Hepatology, University Medical Center Hamburg-Eppendorf, Hamburg, Germany

**Keywords:** Immunology, Innate immune cells, Innate lymphoid cells

## Abstract

In immune-mediated hepatitis, type 2 innate lymphoid cells (ILC2) as well as effector CD4^+^ T cells have been shown to drive disease pathology. However, less is known about mechanisms involved in the regulation of ILC2 function during liver inflammation. We showed that in homeostasis, hepatic ILC2 constituted a very small population with a naive, inactive phenotype. During immune-mediated hepatitis, the cytokines IL-33 and IFNγ were expressed in liver tissue. IL-33 induced strong activation and expression of type 2 cytokines as well as IL-6 by hepatic ILC2 while IFNγ suppressed cytokine production. Interestingly, this inhibitory effect was overcome by IL-33. The phenotype of activated hepatic ILC2 were stable since they did not show functional plasticity in response to liver inflammation-induced cytokines. Moreover, hepatic ILC2 induced a Th2 phenotype in activated CD4^+^ T cells, which increased ILC2-derived cytokine expression via IL-2. In contrast, Th1 cells inhibited survival of ILC2 by production of IFNγ. Thus, hepatic ILC2 function is regulated by IL-33, IL-2, and IFNγ. While IL-33 and IL-2 support hepatic ILC2 activation, their inflammatory activity in immune-mediated hepatitis might be limited by infiltrating IFNγ-expressing Th1 cells.

## Introduction

Innate lymphoid cells (ILC) are a subgroup of lymphocytes characterized by lack of lineage (lin)-specific markers and antigen-specific receptors. Upon activation, ILC rapidly produce effector cytokines and in this way, are involved in the initiation and regulation of immune responses during infection and inflammation^1,2^. ILC are classified into ILC1, ILC2, and ILC3 based on their cytokine and transcription factor expression profile. ILC2 constitute an ILC subset, which is a potent source of type 2 cytokines and predominantly express IL-5, IL-13, and IL-9 in response to activation by IL-33 and IL-25, respectively^3–5^. The activity of ILC2 is inhibited by the inflammatory cytokine IFNγ^6–9^. Moreover, conversion of human ILC2 into ILC1 with concomitant expression of IFNγ has been shown in response to the cytokines IL-1β and IL-12 and termed functional plasticity^10,11^. Beside cytokine-mediated immunity, ILC2 can function as antigen-presenting cells (APC) and induce CD4^+^ T-cell activation and Th2-cell polarization^12,13^.

Dysregulated ILC2 have been described in a variety of diseases such as allergy, asthma, and atopic dermatitis^14^. In the liver, the ILC2-activating cytokine IL-33 acts as an alarmin released in response to hepatic tissue damage. Elevated IL-33 levels were shown in patients with acute and chronic liver disease^15–19^ and therefore, IL-33 has been suggested as a pathogenic factor in hepatic inflammation. In the murine model of concanavalin A (ConA)-induced immune-mediated hepatitis^20^, we have previously shown that effector CD4^+^ T cells cause hepatocyte death by necrosis through production of IFNγ and TNFα^21–23^. Furthermore, the formation of large necrotic lesions was associated with release of IL-33 and activation of ILC2 in the liver, which aggravated disease pathology by activation of inflammatory eosinophils^24^. Several other studies have also been indicated that IL-33-activated ILC2 contribute to the pathogenesis of murine viral hepatitis^25^, biliary carcinogenesis^26^, and liver fibrosis^16^. In addition, in patients with end-stage liver disease, the frequency of hepatic ILC2 has been correlated with disease severity and it was suggested that they contribute to ongoing fibrogenesis^27^. Thus, there is increasing body of evidence that activated hepatic ILC2 exacerbate liver disease pathology. However, mechanisms of ILC2 regulation in liver inflammation are less clear.

In the present study, we investigated the phenotype and plasticity of hepatic ILC2 in response to liver inflammation-induced cytokines and effector CD4^+^ T cells in order to identify mechanisms of ILC2 regulation in liver disease.

## Material and Methods

### Mice

C57BL/6 mice and OT-II mice were bred in the University Medical Center Hamburg-Eppendorf animal facility (Hamburg, Germany). Mouse experiments were conducted according to the German animal protection law and approved by the institutional review board (Behörde für Gesundheit und Verbraucherschutz, Hamburg, Germany; G44/15). Mice received humane care according to the national guidelines of the National Institutes of Health in Germany.

### Animal treatment

C57BL/6 mice received intraperitoneally recombinant murine (rm)IL-33 (0.3 μg; BioLegend, San Diego, CA) daily on four consecutive days. ConA (7 mg/kg; Sigma-Aldrich, St. Louis, MO) was injected intravenously into C57BL/6 mice that were analyzed 8 and 24 hours later. Heart blood was drawn from individual mice and liver injury was quantified by automated measurement of plasma activities of alanine transaminase (ALT) using a COBAS Mira System (Roche Diagnostic, Mannheim, Germany).

### Immunohistochemistry

Liver samples were fixed with 4% formalin and embedded in paraffin. 3 μm liver sections were cut, stained with H&E following standard protocols, and analyzed by light microscopy.

### Isolation of hepatic ILC2 and CD4^+^ T cells

Hepatic non-parenchymal cells were isolated from livers of naive and IL-33-treated mice by Percoll density gradient centrifugation. Single cell suspensions were stained with the Lineage Antibody Cocktail (APC; BD Pharmingen, Heidelberg, Germany) as well as anti-Sca-1 (Pacific Blue; D7; BioLegend) and anti-ST2 (PerCP-eFluor 710; RMST2-2; ThermoFisher Scientific, Waltham, MA) antibodies. Lineage-negative (lin^−^) cells were enriched by magnetic cell sorting and lin^−^ Sca-1^+^ ST2^+^ ILC2 were purely isolated by FACS (BD FACSAriaTM III sorter, BD Biosciences). Ovalbumin (OVA)-specific CD4^+^ T cells were isolated from spleen and lymph nodes of OT-II mice. Therefore, CD4^+^ T cells were enriched from single cell suspensions using the CD4^+^ T Cell Isolation Kit (Miltenyi Biotec, Bergisch Gladbach, Germany). Subsequently, cells were stained with anti-TCRβ (PE-Cy7; H57-597) and anti-CD4 (BV421; GK1.5; both BioLegend) antibodies and TCRβ^+^ CD4^+^ T cells were purified by FACS.

### Th1-cell polarization

OVA-specific CD4^+^ T cells were isolated as described above. Splenic MHCII^+^ cells were isolated by magnetic cell sorting using a PE-conjugated anti-MHCII antibody (2G9; BD Pharmingen) and anti-PE MicroBeads (Miltenyi Biotec). For Th1-cell polarization, CD4^+^ T cells (1 × 10^6^) and MHCII^+^ cells (2 × 10^6^) were co-cultured in the presence of rmIL-2 (10 U/ml), rmIL-12 (10 ng/ml) and OVA_323-339_ peptide (5 µg/ml; ISQAVHAAHAEINEAGR) for 4 days. Thereafter, cultured cells were stained with anti-CD4 (PE; RM4-5) and anti-MHCII (FITC; 2G9; both BioLegend) antibodies and CD4^+^ MHCII^-^ Th1 cells were purified by FACS.

### *In vitro* culture experiments

FACS-isolated hepatic ILC2 (1 × 10^4^) were cultured in the presence of rmIL-33 (10 ng/ml), rmIL-25 (10 ng/ml), rmIL-1β (10–200 ng/ml; all BioLegend), rmIFNγ (10 ng/ml) and rmIL-12 (10–200 ng/ml; both R&D Systems, Wiesbaden, Germany) for 4 days. For ILC2 maintenance, all cultures were done in the presence of rmIL-2 (10 U/ml) and rmIL-7 (10 ng/ml; both R&D Systems). For CD4^+^ T-cell activation, hepatic ILC2 (2 × 10^4^) were co-cultured with FACS-isolated, OVA-specific CD4^+^ T cells (1 × 10^5^) or OVA-specific Th1 cells (1 × 10^5^) in the presence of OVA_323-339_ peptide (5 µg/ml) for 4 days. For blocking IL-2 or IFNγ, co-cultures were done in the presence of an anti-IL-2 (JES-1A12; 10 µg/ml; BD Pharmingen) and anti-IFNγ (R4-6A2; 10 µg/ml; BioXCell, West Lebanon, NH) antibody, respectively.

### Flow cytometry

Cells were incubated with anti-CD16/32 antibody (93; BioLegend) prior to antibody staining in order to prevent unspecific binding. LIVE/DEAD Fixable Staining Kits (Thermo Fisher Scientific) were used to exclude dead cells. For cell surface analysis, cells were stained with the following antibodies: anti-TCRβ (PE-Cy7/PE; H57–597), anti-KLRG1 (PE/BV605; 2F1/KLRG1), anti-CD25 (PE/PE-Cy7; PC-61), anti-CD86 (APC-Cy7; PO3.1), anti-MHCII (FITC; M5/114.15.2; all BioLegend) and anti-CD80 (PE; 16-10A1; ThermoFisher Scientific). For intracellular and intranuclear staining, cells were re-stimulated with phorbol myristate acetate (20 ng/ml) and ionomycin (1 µg/ml) for 6 hours with the addition of brefeldin A (1 µg/ml; all Sigma Aldrich) and monensin (2 µM; BioLegend) after 60 min. After surface and Live/Dead staining, cells were fixed using the Transcription Factor Staining Buffer Set (eBioscience) and incubated in Permeabilization buffer with antibodies specific to IL-2 (PE; JES6-5H4), IL-4 (PerCP-Cy5.5; 11B11), IL-6 (PE; MPS-20F3), TNFα (PE/FITC; MP6-XT22), IFNγ (APC; XMG1.2), GATA3 (FITC; 16E10A23; all BioLegend), IL-5 (PE; TRFK5; BD Pharmingen), and IL-13 (Alexa Flour 488; eBio13A; ThermoFisher Scientific). Data were acquired using a BD LSRFortessa II (BD 172 Biosciences) and analyzed by FlowJo software (Tree Star, Ashland, OR, USA).

### Quantitative real-time PCR analysis

Total RNA was isolated from shock-frozen liver tissue or FACS-sorted hepatic ILC2 using the NucleoSpin RNA Kit (Machery-Nagel, Duren, Germany) and RNeasy Micro Kit (Quiagen, Hilden, Germany), respectively according to the manufacturer’s instruction. RNA was transcribed into cDNA using the Verso cDNA Synthesis Kit (Life Technologies, Carlsbad, CA) on a MyCycler thermal cycler (BioRad, München, Germany). Quantitative RT-PCR was performed using the Absolute qPCR SYBR Green Mixes (Thermo Scientific). The relative mRNA levels were calculated using the ∆∆CT method after normalization to the housekeeping gene GAPDH. Quantification was shown in x-fold changes to the corresponding control cDNA. Primers were obtained from Metabion (Martinsried, Germany). Sequences of the primers are listed in the supporting information.

### Statistical analyses

Data were analyzed using the GraphPad Prism software (GraphPad software, San Diego, CA). Statistical comparison was carried out using the Mann-Whitney U test or the one-way ANOVA with post analysis by Tukey-Kramer test. Data were expressed as means ± SEM. A p value of less than 0.05 was considered statistically significant with the following ranges *p < 0.05, **p < 0.01, ***p < 0.001, and ****p < 0.0001.

## Results

### Hepatic ILC2 exhibit a naive phenotype in homeostasis but potently become activated by the alarmin IL-33

**Figure 1:**
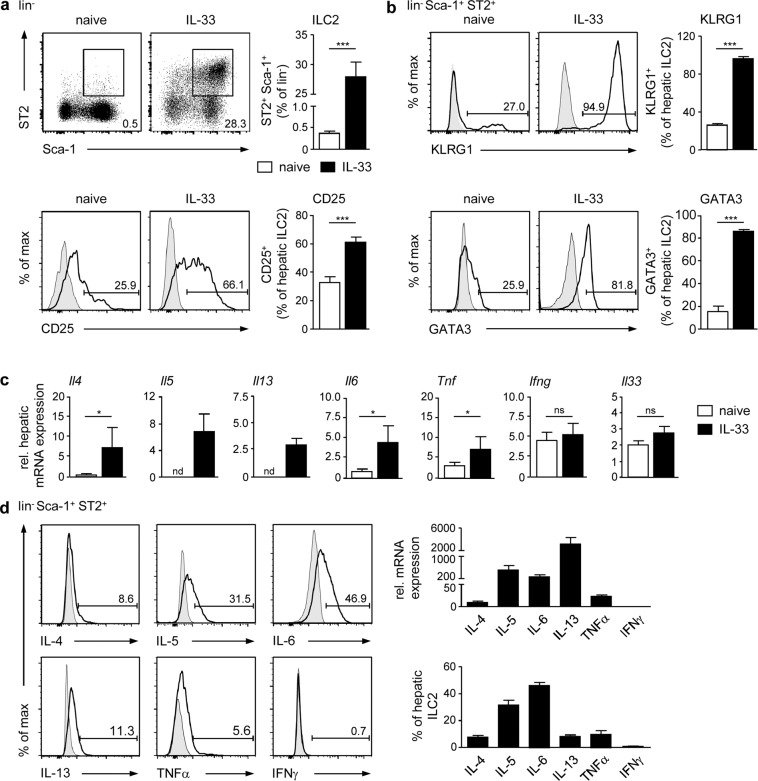
Phenotype of hepatic ILC2 in homeostasis and after IL-33 treatment. C57BL/6 mice were treated with rmIL-33 on four consecutive days. (**a**) Hepatic leukocytes from naive and IL-33-treated mice were stained for lin^−^ Sca-1^+^ ST2^+^ ILC2 and analysed by flow cytometry. Dot plots show frequencies of ILC2 in the liver. (**b**) Hepatic ILC2 were stained for KLRG1, CD25, and GATA3 and analysed by flow cytometry. (**c**) Hepatic mRNA expression was analysed by quantitative RT-PCR in relation to the reference gene GAPDH. (**d**) Hepatic ILC2 were isolated from IL-33-treated mice by FACS. Cytokine mRNA and protein expression were analysed by quantitative RT-PCR in relation to the reference gene GAPDH and by flow cytometry, respectively. Histograms show frequencies of protein-expressing hepatic ILC2. Bold line, antibody staining; filled graph, fluorescence minus one control. Mean ± SEM of 3–4 independent experiments with 3 mice per group are shown. Mann-Whitney U test. *p < 0.05; ***p < 0.001; ns: not significant; nd: not detectable; *Tnf*: tumor necrosis factor-alpha; *Ifng*: interferon-gamma.

Less is known about mechanisms driving activation of ILC2 during liver inflammation. In immune-mediated hepatitis, we have shown previously that formation of large necrotic lesions was associated with release of IL-33 and expansion of ILC2, which expressed the type 2 cytokines IL-5 and IL-13^24^. Since IL-33 has been described as a potent activator of ILC2 in many organs, these data indicate an IL-33-triggered stimulation of ILC2 in immune-mediated hepatitis. However, the phenotype of hepatic ILC2 in homeostasis and after IL-33-induced activation is less clear. Therefore, we performed comparative phenotype analysis of ILC2 from livers of naive mice and mice that were treated with IL-33. We stained for lin^−^ Sca-1^+^ ST2^+^ cells to identify ILC2 in liver tissue (Supplemental Fig. 1a) and determined a small population of liver-resident ILC2 in naive mice (Fig. 1a). In initial kinetic experiments, we found that treatment of mice with IL-33 on four consecutive days was required to induce strong expansion of ILC2 in the liver (Supplemental Fig. 1b, Fig. 1a). In homeostasis, a small frequency of hepatic ILC2 expressed the activation markers killer cell lectin-like receptor G1 (KLRG1) and CD25 as well as the transcription factor GATA3, whose expression was highly elevated in response to four-day IL-33 treatment (Fig. 1b).

We further showed IL-33-induced hepatic mRNA expression of the type 2 cytokines *Il4*, *Il5*, and *Il13* as well as of the pro-inflammatory cytokines *Il6* and tumour necrosis factor alpha (*Tnf*) whereas interferon gamma (*Ifng*) and *Il33* gene expression was not induced. In homeostasis, we did not detect any substantial hepatic type 2 cytokine mRNA expression (Fig. 1c) and also ILC2 did not show any significant cytokine production (data not shown). However, cytokine mRNA and protein expression analysis revealed that IL-33-elicited hepatic ILC2 predominantly expressed IL-5, IL-6, and IL-13. Additionally, a small frequency also expressed IL-4 and TNFα whereas IFNγ was not produced (Fig. 1d). Thus, IL-33 induced strong activation and cytokine response by ILC2 in the liver.

### IL-33 overcomes the inhibitory effect of IFNγ on hepatic ILC2 activity

**Figure 2:**
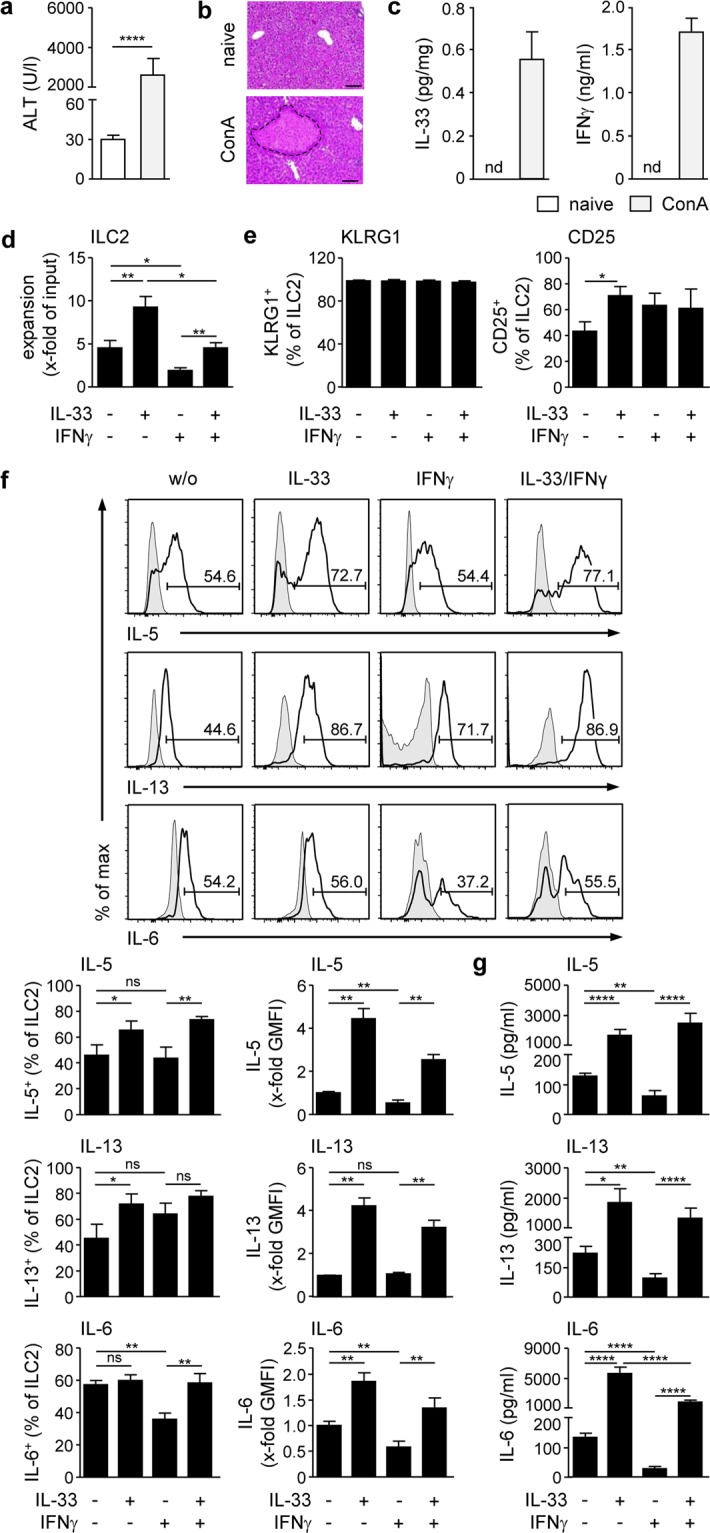
Regulation of hepatic ILC2 function by liver inflammation-induced cytokines. (**a**) C57BL/6 mice received ConA and were analysed 24 hours later. Plasma ALT activity was determined and (**b**) liver samples were stained with H&E to visualize necrotic areas (dotted line). Bars represent 100 µm. (**c**) Relative amount of IL-33 produced during *ex vivo* overnight liver organ cultures was determined by ELISA. Plasma IFNγ levels were determined by ELISA. (**d**) FACS-isolated hepatic ILC2 from IL-33-treated mice were cultured in the presence of IL-33 and/or IFNγ for four days. ILC2 were counted and expansion was calculated in comparison to the input. (**e**) ILC2 were stained for KLRG1 and CD25 and analysed by flow cytometry. (**f**) ILC2 were intracellularly stained for IL-5, IL-13, and IL-6 and analysed by flow cytometry. (**g**) Cytokine levels were determined in culture supernatants by multiplex assay. Histograms show frequency of cytokine-expressing hepatic ILC2. Bold line, antibody staining; filled graph, fluorescence minus one control. Mean ± SEM of 4 independent experiments are shown. (**a**) Mann-Whitney U test; (**d**–**g**) one-way ANOVA with post analysis by Tukey-Kramer test. *p < 0.05; **p < 0.01; ****p < 0.0001; ns: not significant; nd: not detectable; ALT: alanine transferase; ConA: Concanavalin A; w/o: without; GMFI: geometric mean fluorescence intensity.

So far, the regulation of ILC2 function during liver inflammation is insufficiently understood. In ConA-induced immune-mediated hepatitis, liver injury (Fig. 2a) and development of necrosis (Fig. 2b) were associated with up-regulated expression of IL-33 and IFNγ (Fig. 2c). Since these two cytokines have been linked with activation and inhibition of ILC2, respectively, we aimed at investigating the immunomodulatory effect of IL-33 and IFNγ on hepatic ILC2 function *in vitro*. Due to a very low frequency of ILC2 in naive liver (Fig. 1a), which did not allow to isolate hepatic ILC2 in sufficient cell numbers for the *in vitro* studies, we first strongly expanded hepatic ILC2 *in vivo* by treatment of mice with IL-33 on four consecutive days (Fig. 1a, Supplemental Fig. 1b). Thereafter, hepatic lin^−^ Sca-1^+^ ST2^+^ ILC2 were purely isolated by FACS (Supplemental Fig. 1c) and cultured in the presence of IL-33 and/or IFNγ for 4 days. All cultures were done in the presence of IL-2 and IL-7 since without these cytokines, hepatic ILC2 did not survive in the absence of IL-33. We demonstrated that hepatic ILC2 already expanded in the presence of IL-2/IL-7 and expansion was further increased in the presence of IL-33. In contrast, hepatic ILC2 expansion was strongly reduced by IFNγ. Interestingly, the inhibitory effect of IFNγ on hepatic ILC2 expansion was partially overcome by IL-33 (Fig. 2d). IL-33-elicited hepatic ILC2 strongly expressed KLRG1 and to a lesser extent also CD25 (Fig. 1b). High expression of KLRG1 was maintained under all culture conditions whereas the frequency of CD25^+^ ILC2 was significantly increased in the presence of IL-33 (Fig. 2e).

In addition, we analysed cytokine production by hepatic ILC2 in response to IL-33 and/or IFNγ. Both, the frequencies and cellular expression (shown as geometric mean fluorescent intensity (GMFI) of the analysed protein) of IL-5- and IL-13-expressing hepatic ILC2 were strongly increased in the presence of IL-33 (Fig. 2f) resulting in elevated cytokine levels in culture supernatants (Fig. 2g). IL-33 did not enhance the percentage of IL-6-expressing hepatic ILC2 but analysing the GMFI revealed an elevated IL-6 expression resulting in high IL-6 levels in culture supernatants (Fig. 2f,g). IFNγ led to significant reduction in IL-6^+^ ILC2 but did not affect the frequencies of IL-5- and IL-13-expressing hepatic ILC2. However, GMFI and cytokine levels in the culture supernatants of all analysed cytokines were decreased in response to IFNγ. Interestingly, IL-33 induced significantly enhanced production of IL-5, IL-13, and IL-6 by hepatic ILC2 in the presence of IFNγ (Fig. 2f,g). Cytokine multiplex analysis further revealed low production of IL-4 and TNFα by hepatic ILC2, which was not altered by IL-33 whereas IFNγ suppressed expression of TNFα but not IL-4. We also determined low production of IL-9 by IL-33-stimulated hepatic ILC2 that was partially reduced by IFNγ (Supplemental Fig. 2). Under all conditions, hepatic ILC2 did not express IFNγ or IL-17A (data not shown). Thus, hepatic ILC2 function was supported by IL-33 and suppressed by IFNγ. However, IL-33 reversed the inhibitory effect of IFNγ on hepatic ILC2 expansion and cytokine production.

### No functional plasticity of hepatic ILC2 in response to IL-1β/IL-12 but induction of inflammatory ILC2 in response to IL-25

**Figure 3:**
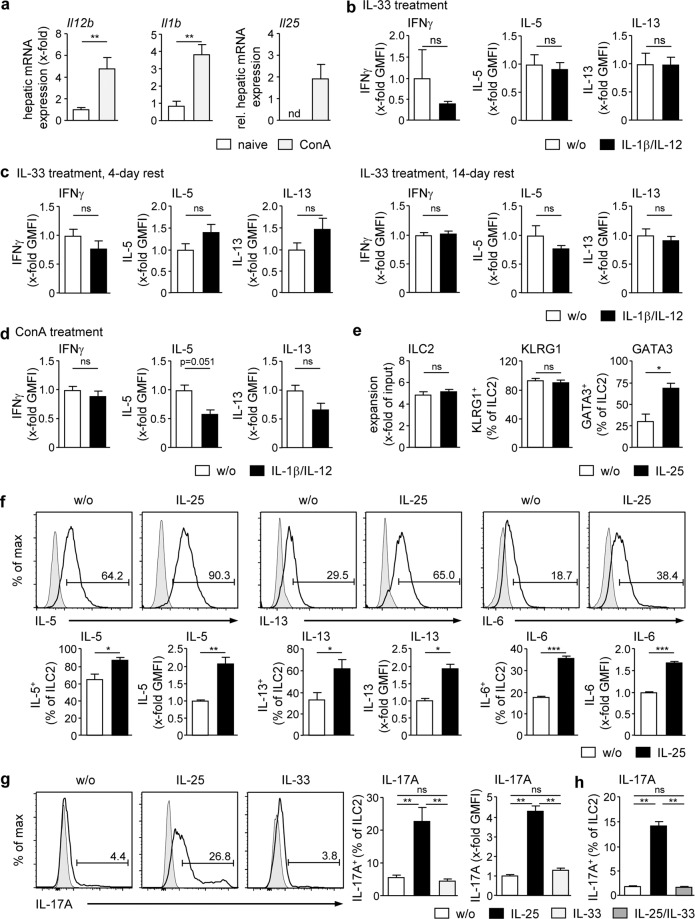
Regulation of hepatic ILC2 function by IL-1β/IL-12 or IL-25. (**a**) C57BL/6 mice received ConA and were analysed 8 hours later. Hepatic mRNA expression was analysed by quantitative RT-PCR and normalized to naive mice. (**b**) FACS-isolated hepatic ILC2 from IL-33-treated mice were cultured in the presence of IL-1β/IL-12 for 4 days. (**c**) Hepatic ILC2 were cultured in the presence of IL-2/IL-7 for 4 or 14 days. Thereafter, IL-1β and IL-12 were added and ILC2 were cultured for 4 further days. (**d**) Hepatic ILC2 were isolated from ConA-treated mice 24 h after induction of immune-mediated hepatitis and cultured in the presence of IL-1β/IL-12 for 4 days. GMFIs of IFNγ-, IL-5- or IL-13-expressing ILC2 were determined by flow cytometry and normalized to GMFIs of ILC2 cultured in the absence of IL-1β/IL-12. (**e–h**) Hepatic ILC2 were cultured in the presence of IL-25 and/or IL-33 for 4 days and analysed by flow cytometry. Histograms show frequency of cytokine-expressing hepatic ILC2. Bold line, antibody staining; filled graph, fluorescence minus one control. Mean ± SEM of 2–3 independent experiments are shown. (**a–f**) Mann-Whitney U test; (**g,h**) one-way ANOVA with post analysis by Tukey-Kramer test. *p < 0.05; **p < 0.01; ***p < 0.001; ns: not significant; nd: not detectable; GMFI: geometric mean fluorescence intensity, ConA: Concanavalin A; w/o: without.

Conversion of ILC2 into IFNγ-expressing ILC1 with concomitant reduction of IL-5 and IL-13 expression was shown in response to the cytokines IL-1β and IL-12 and termed functional plasticity^10,11^. In addition, ‘inflammatory’ ILC2 have been described, which start to express the pro-inflammatory cytokine IL-17A in response to IL-25^28^. Since hepatic gene expression of *Il1b*, *Il12b* and *Il25* was induced during immune-mediated hepatitis (Fig. 3a), we asked whether hepatic ILC2 can convert their phenotype in response to these liver inflammation-induced cytokines. To analyse functional plasticity, hepatic ILC2 from IL-33-treated mice were cultured in the presence of IL-1β and IL-12 for 4 days. We neither determined induction of IFNγ expression in hepatic ILC2 nor decreased IL-5 and IL-13 expression (Fig. 3b). Hence, we tested increasing concentrations of IL-1β and IL-12, however, hepatic ILC2 did not start to express IFNγ (Supplemental Fig. 3a,b). In addition, resting hepatic ILC2, which were cultured in the presence of IL-2/IL-7 for 4 days and 14 days, respectively, before stimulation with IL-1β/IL-12, did not show induction of IFNγ expression or down-regulation of IL-5 and IL-13 expression (Fig. 3c). We also isolated hepatic ILC2 from ConA-treated mice and stimulated these cells with IL-1β/IL-12 but again, expression of IFNγ was not induced by these cytokines (Fig. 3d).

We further analysed the ability of IL-25 to induce inflammatory hepatic ILC2. Therefore, ILC2 were cultured in the presence of IL-25 for 4 days. We showed that IL-25 did not induce substantial expansion of hepatic ILC2, whereas high KLRG1 expression was maintained. Similar to IL-33, IL-25 enhanced expression of GATA3, IL-5, IL-13, and IL-6 by hepatic ILC2 (Fig. 3e,f). Interestingly, IL-25 induced expression of IL-17A by hepatic ILC2, which was not determined after stimulation with IL-33 (Fig. 3g). However, IL-25-induced IL-17A expression was inhibited in the presence of IL-33 (Fig. 3h). Thus, hepatic ILC2 showed no functional plasticity in response to IL-1β/IL-12 but started to express IL-17A in response to IL-25.

### ILC2-activated CD4^+^ T cells increase their cytokine expression by production of IL-2

**Figure 4:**
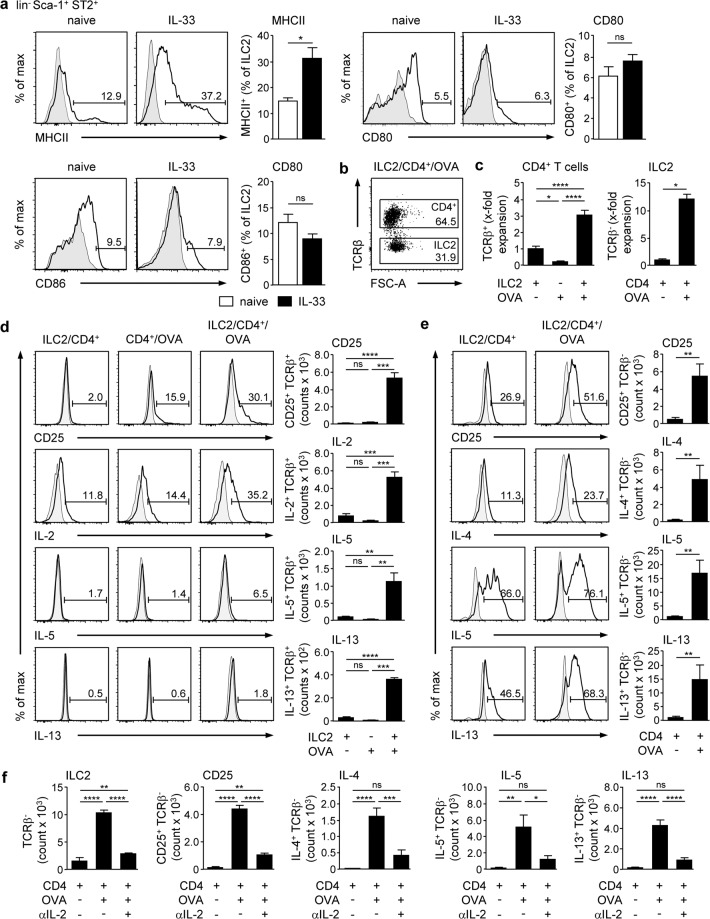
Antigen-specific cross-talk of hepatic ILC2 and CD4^+^ T cells. (**a**) C57BL/6 mice were treated with IL-33 on four consecutive days. Hepatic ILC2 from naive and IL-33-treated mice were stained for MHCII, CD80, and CD86 and analysed by flow cytometry. (**b**) Hepatic ILC2 from IL-33-treated mice were co-cultured with OVA-specific CD4^+^ T cells from OT-II mice in presence or absence of OVA for 4 days. Phenotype analysis was done in TCRβ^+^ CD4^+^ T cells and TCRβ^-^ ILC2. (**c**) Cells were counted and expansion was calculated in comparison to the co-culture without OVA. (**d**) Activation and intracellular cytokine expression were analysed in CD4^+^ T cells and (**e**) hepatic ILC2 by flow cytometry. (**f**) Co-cultures of hepatic ILC2 and CD4^+^ T cells were done in presence or absence of OVA and an anti-IL-2 antibody for 4 days. Dot plot shows frequencies of CD4^+^ T cells and ILC2. Histograms show frequencies of cytokine-expressing hepatic ILC2 and CD4^+^ T cells. Bold line, antibody staining; filled graph, fluorescence minus one control. Mean ± SEM of 2–3 independent experiments are shown. (**a,c,e**) Mann-Whitney U test; (**c,d,f**) one-way ANOVA with post analysis by Tukey-Kramer test. *p < 0.05; **p < 0.01; ***p < 0.001; ****p < 0.0001; ns: not significant.

In immune-mediated hepatitis, CD4^+^ T cells infiltrate the liver and contribute to disease pathology^20,21,23^. We analysed whether hepatic ILC2 and CD4^+^ T cells can impact each other *in vitro* and what the outcome of such an interaction is. We showed that hepatic ILC2 expressed MHCII and the co-stimulatory molecules CD80/CD86 (Fig. 4a), indicating that they have the potential for CD4^+^ T-cell activation. To study activation of CD4^+^ T cells, ovalbumin (OVA)-specific CD4^+^ T cells were isolated from spleen and lymph nodes of naive OT-II mice by FACS and co-cultured with hepatic ILC2 in the presence of OVA for 4 days. For co-culture experiments, we isolated hepatic ILC2 from mice that were treated with IL-33 on four consecutive days resulting in increased expression of MHCII while expression of co-stimulatory molecules was not altered (Fig. 4a). Since IL-2 and IL-7 induce expansion of hepatic ILC2 (Fig. 2d), all co-cultures were done without these cytokines. In flow cytometry analysis, we distinguished TCRβ^+^ CD4^+^ T cells from TCRβ^-^ ILC2 (Fig. 4b). We counted total cell numbers after co-culture and calculated the respective numbers of CD4^+^ T cells and ILC2 based on the frequencies of both cell populations, which we determined by flow cytometry (Fig. 4b). Thereafter, we calculated the ratio between the number of CD4^+^ T cells or ILC2 after co-culture and the respective cell numbers before co-culture and depicted the data as x-fold expansion (Fig. 4b,c). We showed expansion of CD4^+^ T cells in the presence of ILC2 and OVA whereas CD4^+^ T cells that were cultured with either ILC2 or antigen alone did not expand (Fig. 4c). Frequency and number of CD4^+^ T cells expressing the activation marker CD25 as well as the cytokines IL-2 and IL-13 were elevated after co-culture with ILC2 and the antigen while IL-13 expression was only slightly induced (Fig. 4d). We did not detect expression of inflammatory cytokines like IFNγ or anti-inflammatory cytokines like IL-10 by hepatic ILC2-activated CD4^+^ T cells (data not shown).

Interestingly, expansion (Fig. 4c), activation and type 2 cytokine production of hepatic ILC2 were enhanced in the presence of activated CD4^+^ T cells (Fig. 4e), while ILC2 did not survive if cultured without CD4^+^ T cells (data not shown). Since ILC2 express the IL-2 receptor but did not produce substantial amounts of IL-2 (data not shown), we analysed whether CD4^+^ T cell-derived IL-2 triggered hepatic ILC2 activation. Therefore, we performed co-cultures in the presence of an anti-IL-2 antibody. We showed that blockage of IL-2 abrogated expansion of ILC2 and also expression of CD25, IL-4, IL-5, and IL-13 in the presence of activated CD4^+^ T cells (Fig. 4f). Thus, hepatic ILC2-activated CD4^+^ T cells promote their activation and type 2 cytokine production by expression of IL-2.

### Th1 cells inhibit hepatic ILC2 survival by production of IFNγ

**Figure 5:**
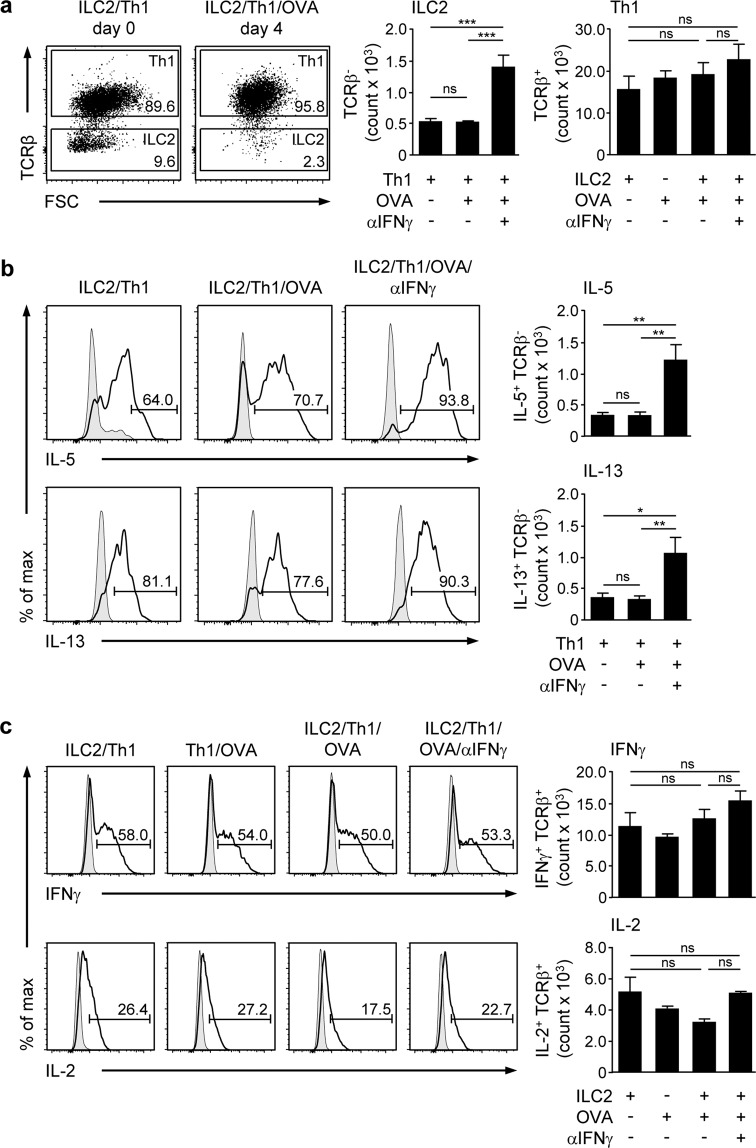
Effects of Th1 cells on hepatic ILC2 function. Hepatic ILC2 from IL-33-treated mice were co-cultured with *in-vitro* polarized OVA-specific Th1 cells in the presence of OVA and an anti-IFNγ antibody for 4 days. (**a**) Frequency and number of TCRβ^+^ Th1 cells and TCRβ^-^ ILC2 were determined by flow cytometry. (**b**) Intracellular cytokine expression was analysed in hepatic ILC2 and (**c**) Th1 cells by flow cytometry. Dot plots show frequencies of Th1 cells and ILC2. Histograms show frequencies of cytokine-expressing hepatic ILC2 and Th1 cells. Bold line, antibody staining; filled graph, fluorescence minus one control. Mean ± SEM of 2 independent experiments are shown. One-way ANOVA with post analysis by Tukey-Kramer test. *p < 0.05; **p < 0.01; ***p < 0.001; ns: not significant.

Th1 cells are one effector T-cell population that infiltrate the liver during immune-mediated hepatitis^21,22^. Since Th1 cells produce IFNγ, we studied their effect on hepatic ILC2 activity and function *in vitro*. To polarize Th1 cells, CD4^+^ T cells from OT-II mice were co-cultured with splenic MHCII^+^ cells in the presence of IL-2, IL-12, and OVA for 4 days. Thereafter, MHCII^+^ cells were depleted by FACS (Supplemental Fig. 4a). We showed strong expression of IFNγ and lack of IL-5, IL-13, and IL-17A expression in the cultured CD4^+^ T cells thereby confirming proper Th1-cell polarisation (Supplemental Fig. 4b). These Th1 cell were co-cultured with hepatic ILC2 in absence or presence of OVA for 4 days. To block IFNγ, co-cultures were done in the presence of an anti-IFNγ antibody. We determined a very low frequency and number of hepatic ILC2 after co-culture with Th1 cells that were not altered in the presence of the antigen (Fig. 5a). However, blockage of IFNγ enhanced the frequency of living, IL-5 and IL-13-expressing ILC2 (Fig. 5b). Hepatic ILC2 were not able to re-activate or modulate Th1 cells since neither number nor cytokine expression were changed after co-culture with ILC2 and OVA (Fig. 5a,c). Moreover, the positive effect of IFNγ blockage on ILC2 survival and cytokine production did also not affect the phenotype of Th1 cells (Fig. 5c). Thus, hepatic ILC2 have no impact on Th1 cells and instead were inhibited through Th1 cell-derived IFNγ.

## Discussion

ILC2 have been described as a tissue-resident cell population whose function is mainly regulated by the local cytokine milieu, which depends on the organ and the type of initiated immune response. Although there are some studies addressing the role of ILC2 in the pathogenesis of liver diseases, mechanisms of hepatic ILC2 activation and regulation are less clear. In this study, we described molecular and cellular pathways that might be involved in the regulation of hepatic ILC2 function during liver inflammation.

We showed that among hepatic leukocytes, ILC2 constituted a very small population in liver homeostasis that was characterized by low expression of KLRG1, CD25, and GATA3 thereby resembling naive ILC2^29^. Elevated IL-33 levels have been shown in patients with acute and chronic liver inflammation^15–19^, suggesting that this cytokine plays a role in liver disease pathology. Therefore, we studied the effect of IL-33 on ILC2 phenotype and function in the liver. We found that the hepatic ILC2 population strongly expanded during four-day IL-33 treatment and highly up-regulated expression of KLRG1, CD25, and GATA3. Given that KLRG1 and CD25 have been identified as markers of mature and activated ILC2^30,31^, and GATA3 was shown to be required for ILC2 maintenance and function^30–33^, these data identified IL-33 as a potent activator of hepatic ILC2 *in vivo*. Moreover, ILC2 have been described as producers of type 2 cytokines upon activation. In line with this, IL-33-stimulated hepatic ILC2 predominantly expressed IL-5, and to a lesser extent also IL-13 and IL-4. There are some studies correlating inflammation-induced hepatic IL-33 expression with activation of ILC2 in the liver^16,24,25^. During chronic liver damage, elevated IL-33 levels were associated with expansion of ILC2 expressing IL-13 thereby activating hepatic stellate cells, the key drivers of liver fibrosis^16^. Increased hepatic IL-33 expression accompanied by expansion of ILC2 were also demonstrated in viral hepatitis^25^ and immune-mediated hepatitis^24^. We could show that activation of hepatic ILC2 was associated with IL-5 expression and infiltration of eosinophils into the liver. Based on these findings, we have suggested that ILC2-derived IL-5 contributes to activation and recruitment of inflammatory eosinophils thereby promoting liver inflammation and tissue damage in immune-mediated hepatitis^24^. Interestingly, we identified IL-6 as the main cytokine expressed by IL-33-activated hepatic ILC2. However, the role of ILC2-derived IL-6 for liver disease pathology is not clear. In general, IL-6 is a cytokine with beneficial effects for the liver. It has been shown that IL-6 promotes liver regeneration^34^, induces expression of anti-apoptotic proteins^35,36^ and dampens pathology of hepatic ischemia/reperfusion injury^37^, alcohol- and CCl_4_-induced liver injury^36,38^. In immune-mediated hepatitis, both pro- and anti-inflammatory functions of IL-6 have been described. While *Il6*^−/−^ mice developed less severe immune-mediated hepatitis^39,40^, IL-6 pre-treatment before induction of hepatitis^41^ as well as IL-6 production induced by a soyasapogenol derivative ameliorated liver injury^42^. Thus, IL-6 plays an important role in the pathology of liver diseases and ILC2 might contribute to IL-6-mediated hepatic immune regulation following activation by IL-33.

So far, mainly inflammatory effects of ILC2 on liver disease pathology have been described. It is therefore important to identify mechanisms that might dampen the activity of hepatic ILC2 in order to avoid excessive liver inflammation and tissue damage. In this context, IFNγ has been identified as a suppressor of ILC2 function. Since this cytokine is also expressed in immune-mediated hepatitis, we studied the effects of IFNγ on hepatic ILC2 activity. While IL-33 further increased hepatic ILC2 expansion and cytokine expression *in vitro*, IFNγ showed the opposite effect. We determined a reduced expansion of hepatic ILC2 as well as IL-5, IL-13, and IL-6 expression if IFNγ was present, strongly indicating that IFNγ inhibits hepatic ILC2 function. These findings are in line with previous studies showing that lung ILC2 were inhibited by IFNγ *in vitro*^6^ and *in vivo*^7^, and that in *Ifngr1*^−/−^ mice, ILC2-mediated lung inflammation was aggravated^6^. In contrast, Yeti mice, characterized by constitutive high expression of IFNγ, showed decreased numbers of cytokine-expressing ILC2 and a reduced capacity for parasite clearance^7^. Interestingly, we found that IL-33 abrogated the inhibitory effect of IFNγ, suggesting that in the presence of IL-33, the IFNγ-mediated inhibition of hepatic ILC2 activity is suppressed.

One limitation of these *in vitro* studies is the initial IL-33-mediated *in vivo* expansion of hepatic ILC2 to get sufficient cell numbers for the experiments. As a consequence, all *in vitro* cultures were done with pre-activated hepatic ILC2 and therefore, might not fully reflect the *in vivo* situation. However, we observed similar effects of IL-33 on naive ILC2 *in vivo* and pre-activated hepatic ILC2 used for *in vitro* experiments. In both cases, IL-33 increased expression of IL-5, IL-13, and IL-6 by hepatic ILC2. Moreover, the inhibitory effect of IFNγ on hepatic ILC2 activity and cytokine expression was shown in pre-activated ILC2. Whether IFNγ has any effect on naive hepatic ILC2 is not clear. However, they have an inactive phenotype and do not express cytokines in liver homeostasis and thus, might be unaffected by IFNγ. Another limitation of the *in vitro* studies is the presence of IL-2 and IL-7 in culture medium to ensure survival of hepatic ILC2 that were not stimulated by IL-33 *in vitro*. IL-2 and IL-7 induced expansion of hepatic ILC2 independently of IL-33. Nevertheless, the additional presence of IL-33 further increased their expansion and cytokine expression while IFNγ suppressed both. With regard to IFNγ, it might be conceivable that its inhibitory effect on hepatic ILC2 activity would be more pronounced in absence of IL-2 and IL-7.

Beside IL-33 and IFNγ, IL-12 and IL-1β were also expressed in the inflamed liver during immune-mediated hepatitis. Since these cytokines have been shown to induce differentiation of ILC2 into IFNγ-producing ILC1^10,11^, termed as functional plasticity, we studied their effects on hepatic ILC2 cytokine expression. We tested hepatic ILC2 from both IL-33- and ConA-treated mice but neither determined induction of IFNγ expression nor down-regulation of IL-5- and IL-13 expression in response to IL-12 and IL-1β. These data suggest that functional plasticity is not induced in hepatic ILC2 during immune-mediated hepatitis and that hepatic ILC2 do not drive liver inflammation by conversion into ILC1 expressing the inflammatory cytokine IFNγ.

We further showed induction of hepatic IL-25 expression in immune-mediated hepatitis. IL-25 was found to induce type 2 cytokine expression^43,44^, and in line with this, IL-25-stimulated hepatic ILC2 strongly increased expression of IL-5 and IL-13, but also IL-6. In contrast to IL-33, IL-25 also induced expression of IL-17A by hepatic ILC2. In previous studies, IL-25-responsive IL-17A^+^ ILC2 have been described as inflammatory ILC2 that arise in the course of inflammation^28,45^. In contrast to natural ILC2, inflammatory ILC2 are not present in homeostasis and characterized by expression of the IL-25/IL-17B receptor IL-17RB and lack of ST2^45^. However, we identified and purified hepatic ILC2 by expression of ST2 and this ST2^+^ ILC2 population responded to IL-25 by both, production of type 2 cytokines as well as IL-17A. Moreover, we demonstrated that IL-33 has an inhibitory effect on IL-25-mediated IL-17A expression in hepatic ILC2, indicating that in IL-33-triggered liver diseases, the development of inflammatory ILC2 might be suppressed.

CD4^+^ T cells play an important role in the pathology of liver diseases like autoimmune hepatitis^46^ and therefore, we wondered whether there might be an interaction between hepatic ILC2 and CD4^+^ T cells, which could influence the activity of both cell populations. After activation, CD4^+^ T cells produce effector cytokines that can affect ILC2 activity. In this context, it has been proposed that ILC2 function as APC thereby inducing CD4^+^ T-cell activation and Th2-cell polarization^12,13,47,48^. We found that hepatic ILC2 expressed molecules linked with APC function. We identified a crosstalk between hepatic ILC2 and CD4^+^ T cells, in which ILC2 induced activation of CD4^+^ T cells that in turn produce IL-2 thereby promoting ILC2 function. IL-2 was the main cytokine expressed by hepatic ILC2-activated CD4^+^ T cells. We further detected a low frequency of IL-5- and IL-13-expressing CD4^+^ T cells, indicating that hepatic ILC2 have only a minor capacity to induce Th2-cell polarization. Interestingly, the most striking effect of the crosstalk was on hepatic ILC2 activity. Here, we showed strong ILC2 expansion and elevated type 2 cytokine expression. This was dependent on IL-2 produced by CD4^+^ T cells. We showed that CD4^+^ T cells, which were activated by hepatic ILC2, expressed IL-2 whereas non-activated CD4^+^ T cells expressed only low amounts of this cytokine. Since hepatic ILC2 did not express IL-2, they depend on CD4^+^ T cell-derived IL-2 for survival and expansion. As a consequence, hepatic ILC2 cultured without CD4^+^ T cells did not survive. In the presence of non-activated CD4^+^ T cells, hepatic ILC2 survived most likely due to low IL-2 expression by CD4^+^ T cells. However, we only observed expansion of hepatic ILC2 in the presence of activated CD4^+^ T cells, which strongly increased expression of IL-2. Blockage of IL-2 abrogated hepatic ILC2 survival, activation and cytokine production further demonstrating the importance of IL-2 for ILC2 maintenance in absence of activating cytokines like IL-33. By using an anti-IL-2 antibody, CD4^+^ T cell-activation was abrogated trough preventing IL-2-mediated autocrine self-stimulation. Both, blocking secreted IL-2 and abrogation of full CD4^+^ T-cell activation subsequently caused low IL-2 levels in the co-culture system thereby suppressing hepatic ILC2 activation, expansion and cytokine expression. In contrast to IL-33, which did not induce substantial IL-4 expression, CD4^+^ T-cell-derived IL-2 increased expression of IL-4 by hepatic ILC2. Such an antigen-specific ILC2/CD4^+^ T-cell interaction has also been described in previous studies and was found to be essential for effective intestinal parasitic clearance^12^, and for triggering CD4^+^ T-cell responses to antigen in the lung^48^. Whether this has also relevance for liver disease pathology is still an open question and needs to be further addressed. This would include development of suitable *in vivo* models, e.g. a mouse line, in which ILC2 specifically do not express MHCII. However, it is tempting to speculate that during hepatitis, IL-2 produced by liver-infiltrating T cells might support effector function of hepatic ILC2.

Th1 cells are characterized by high expression of IFNγ and it was shown that they infiltrate the liver during immune-mediated hepatitis^21,22^. Therefore, we were interested in the impact of Th1 cells on hepatic ILC2 activity and function. We showed a strong inhibitory effect of Th1 cells on hepatic ILC2. They were not able to re-activate Th1 cells and instead died. Besides IFNγ, Th1 cells also expressed IL-2 demonstrating that in the presence of IFNγ, IL-2 did not support hepatic ILC2 function. Blockage of IFNγ promoted ILC2 survival and cytokine response, indicating that Th1 cell-derived IFNγ induced hepatic ILC2 death. This occurred independently of antigen presentation, suggesting that still the presence of Th1 cells in proximity to hepatic ILC2 restricts their survival and effector function. Such an inhibitory effect of T cell-derived IFNγ was also shown for renal ILC2 in murine systemic lupus erythematosus^9^. Thus, the inflammatory activity of hepatic ILC2 in liver disease might be limited by liver-infiltrating Th1 cells.

In summary, this study revealed potent activation of hepatic ILC2 by IL-33 and CD4^+^ T cell-derived IL-2 whereas IFNγ, e.g. expressed by Th1 cells, inhibits their survival and function. Considering that IL-33 activates hepatic ILC2, which drive pathology of acute and chronic liver disease, and counteracts the inhibitory effect of IFNγ, treatment strategies targeting IL-33 expression during liver inflammation might prevent hepatic ILC2 activation and promote their inhibition.

## Supplementary information

Supplementary information.

## Data Availability

The datasets generated during and/or analyzed during the current study are available from the corresponding author on reasonable request.

## References

[1] Diefenbach A, Colonna M, Koyasu S (2014). Development, differentiation, and diversity of innate lymphoid cells. Immunity..

[2] Sonnenberg GF, Artis D (2015). Innate lymphoid cells in the initiation, regulation and resolution of inflammation. Nat. Med..

[3] Moro K (2010). Innate production of TH2 cytokines by adipose tissue-associated c-Kit+ Sca-1+ lymphoid cells. Nature..

[4] Neill DR (2010). Nuocytes represent a new innate effector leukocyte that mediates type-2 immunity. Nature..

[5] Wilhelm C (2011). An IL-9 fate reporter demonstrates the induction of an innate IL-9 response in lung inflammation. Nat. Immunol..

[6] Moro K (2016). Interferon and IL-27 antagonize the function of group 2 innate lymphoid cells and type 2 innate immune responses. Nat. Immunol..

[7] Molofsky AB (2015). InterleuKin-33 And Interferon-Γ Counter-Regulate Group 2 Innate Lymphoid Cell Activation During Immune Perturbation. Immunity..

[8] Califano D (2018). IFN-γ increases susceptibility to influenza A infection through suppression of group II innate lymphoid cells. Mucosal Immunol..

[9] Düster M (2018). T cell-derived IFN-γ downregulates protective group 2 innate lymphoid cells in murine lupus erythematosus. Eur. J. Immunol..

[10] Ohne Y (2016). IL-1 is a critical regulator of group 2 innate lymphoid cell function and plasticity. Nat. Immunol..

[11] Lim AI (2016). IL-12 drives functional plasticity of human group 2 innate lymphoid cells. J. Exp. Med..

[12] Oliphant CJ (2014). MHCII-mediated dialog between group 2 innate lymphoid cells and CD4+ T cells potentiates type 2 immunity and promotes parasitic helminth expulsion. Immunity..

[13] Drake LY, Iijima K, Kita H (2014). Group 2 innate lymphoid cells and CD4+ T cells cooperate to mediate type 2 immune response in mice. Allergy..

[14] Bernink JH, Germar K, Spits H (2014). The role of ILC2 in pathology of type 2 inflammatory diseases. Curr. Opin. Immunol..

[15] Roth GA (2010). Up-regulation of interleukin 33 and soluble ST2 serum levels in liver failure. J. Surg. Res..

[16] Mchedlidze T (2013). Interleukin-33-dependent innate lymphoid cells mediate hepatic fibrosis. Immunity..

[17] Wang J (2012). Serum IL-33 levels are associated with liver damage in patients with chronic hepatitis B. J. Interferon Cytokine Res..

[18] Wang J (2012). Serum IL-33 levels are associated with liver damage in patients with chronic hepatitis C. Mediators Inflamm..

[19] Marvie P (2010). Interleukin-33 overexpression is associated with liver fibrosis in mice and humans. J. Cell. Mol. Med..

[20] Tiegs G, Hentschel J, Wendel A (1992). A T cell-dependent experimental liver injury in mice inducible by concanavalin A. J. Clin. Invest..

[21] Kusters S, Gantner F, Kunstle G, Tiegs G (1996). Interferon gamma plays a critical role in T cell-dependent liver injury in mice initiated by concanavalin A. Gastroenterology..

[22] Robinson RT (2009). End-organ damage in a mouse model of fulminant liver inflammation requires CD4+ T cell production of IFN-gamma but is independent of Fas. J. Immunol..

[23] Gantner F, Leist M, Lohse AW, Germann PG, Tiegs G (1995). Concanavalin A-induced T-cell-Mediated hepatic injury in mice: The role of tumor necrosis factor. Hepatology..

[24] Neumann K (2016). A Proinflammatory Role of Type 2 Innate Lymphoid Cells in Murine Immune-Mediated Hepatitis. J. Immunol..

[25] Liang Y (2013). IL-33 Induces Nuocytes and Modulates Liver Injury in Viral Hepatitis. J. Immunol..

[26] Nakagawa H (2017). Biliary epithelial injury-induced regenerative response by IL-33 promotes cholangiocarcinogenesis from peribiliary glands. Proc. Natl. Acad. Sci..

[27] Jeffery HC (2017). Human intrahepatic ILC2 are IL-13positiveamphiregulinpositiveand their frequency correlates with model of end stage liver disease score. PLoS One..

[28] Huang Y (2015). IL-25-responsive, lineage-negative KLRG1 hi cells are multipotential “inflammatory” type 2 innate lymphoid cells. Nat. Immunol..

[29] Li B (2019). T cells and ILC2s are major effector cells in influenza-induced exacerbation of allergic airway inflammation in mice. Eur. J. Immunol..

[30] Hoyler T (2018). The Transcription Factor GATA-3 Controls Cell Fate and Maintenance of Type 2 Innate Lymphoid Cells. Immunity..

[31] Li BWS (2017). Group 2 innate lymphoid cells exhibit a dynamic phenotype in allergic airway inflammation. Front. Immunol..

[32] Yagi R (2014). The transcription factor GATA3 is critical for the development of all IL-7Rα-expressing innate lymphoid cells. Immunity..

[33] Klein Wolterink RGJ (2013). Essential, dose-dependent role for the transcription factor Gata3 in the development of IL-5+ and IL-13+ type 2 innate lymphoid cells. Proc. Natl. Acad. Sci..

[34] Taub R (2004). Liver regeneration: From myth to mechanism. Nat. Rev. Mol. Cell. Biol..

[35] Kovalovich K (2001). Interleukin-6 Protects against Fas-mediated Death by Establishing a Critical Level of Anti-apoptotic Hepatic Proteins FLIP, Bcl-2, and Bcl-xL. J. Biol. Chem..

[36] Hong F (2002). Elevated interleukin-6 during ethanol consumption acts as a potential endogenous protective cytokine against ethanol-induced apoptosis in the liver: Involvement of induction of bcl-2 and bcl-xlproteins. Oncogene..

[37] Camargo CA, Madden JF, Gao W, Selvan RS, Clavien PA (1997). Interleukin-6 protects liver against warm ischemia/reperfusion injury and promotes hepatocyte proliferation in the rodent. Hepatology..

[38] Kovalovich K (2000). Increased toxin-induced liver injury and fibrosis in interleukin-6- deficient mice. Hepatology..

[39] Malchow S (2011). Essential role of neutrophil mobilization in concanavalin A-induced hepatitis is based on classic IL-6 signaling but not on IL-6 trans-signaling. Biochim. Biophys. Acta - Mol. Basis. Dis..

[40] Cao Q, Batey R, Pang G, Russell A, Clancy R (1998). IL-6, IFN-γ and TNF-α production by liver-associated T cells and acute liver injury in rats administered concanavalin A. Immunol. Cell. Biol..

[41] Mizuhara H (1994). T cell activation-associated hepatic injury: mediation by tumor necrosis factors and protection by interleukin 6. J. Exp. Med..

[42] Klein C (2003). ME3738 protects from concanavalin A-induced liver failure via an IL-6-dependent mechanism. Eur. J. Immunol..

[43] Fort MM (2001). IL-25 Induces IL-4, IL-5, and IL-13 and Th2-associated pathologies *in vivo*. Immunity..

[44] Mjösberg JM (2001). Human IL-25-and IL-33-responsive type 2 innate lymphoid cells are defined by expression of CRTH2 and CD161. Nat. Immunol..

[45] Huang Y, Paul WE (2016). Inflammatory group 2 innate lymphoid cells. Int. Immunol..

[46] Liberal R, Grant CR, Mieli-Vergani G, Vergani D (2013). Autoimmune hepatitis: A comprehensive review. J. Autoimmun..

[47] Neill DR (2010). Nuocytes represent a new innate effector leukocyte that mediates type-2 immunity. Nature..

[48] Mirchandani AS (2014). Type 2 Innate Lymphoid Cells Drive CD4+ Th2 Cell Responses. J. Immunol..

