# Supplementary material for: Hepatic ILC2 activity is regulated by liver inflammation-induced cytokines and effector CD4+ T cells
Source: Sci Rep. 2020 Jan 23;10:1071. doi: 10.1038/s41598-020-57985-w (PMC6978388; doi:10.1038/s41598-020-57985-w)
Supplement: Supplementary file 1 — Supplementary information. [file 41598_2020_57985_MOESM1_ESM.pdf]

SUPPLEMENTARY MATERIAL

Hepatic ILC2 activity is regulated by liver inflammation-induced cytokines and effector CD4$^+$ T cells

Silja Steinmann[*1], Marek Schoedsack[*1], Fabian Heinrich[*], Philippe C. Breda[*], Aaron Ochel[*], Gisa Tiegs[*], Katrin Neumann[*]

[*]Institute of Experimental Immunology and Hepatology, University Medical Center Hamburg-Eppendorf, Hamburg, Germany

[1]S.S. and M.S. contributed equally to this work.

Table of content: 1 table, 4 figures

Table 1. Sequences of the primer used for analysis of mRNA expression.

**Target**	**Forward primer**<br>**Reverse primer**	**Amplicon length**	**Annealing temperature**
GAPDH	5´-ACCCTTAAGAGGGATGCTGC-3´<br>3´-CCCAATACGGCCAAATCCGT-5´	136 bp	60°C
IL-1β	5´- GCCACCTTTTGACAGTGATGAG-3´<br>3´-GACAGCCCAGGTCAAAGGTT-5´	95 bp	60°C
IL-4	5´-TCAACCCCCAGCTAGTTGTC-3´<br>3´-AAATATGCGAAGCACCTTGG-5´	227 bp	60°C
IL-5	5´-ATGGAGATTCCCATGAGCAC-3´<br>3´-CCCACGGACAGTTTGATTCT-5´	180 bp	58°C
IL-6	5´-GATGGATGCTACCAAACTGGA-3´<br>3´-GGAAATTGGGGTAGGAAGGA-5´	222 bp	60°C
IL-12p40	5´-AGGTCACACTGGACCAAAGG-3´<br>3´-TGGTTTGATGATGTCCCTGA-5´	173 bp	60°C
IL-13	5´-CTTGCTTGCCTTGGTGGTCT-3´<br>3´-CACAGGGGAGTCTGGTCTTG-5´	122 bp	60°C
IL-25	5´-GAGGAGTGGCTGAAGTGGAG-3´<br>3´-CATGTGGGAGCCTGTCTGTA-5´	228 bp	60°C
IL-33	5´-ATGGGAAGAAGCTGATGGTG-3´<br>3´-CCGAGGACTTTTTGTGAAGG-5´	150 bp	58°C
IFNγ	5´-GAACGCTACACACTGCATC-3´<br>3´-GAGCTCATTGAATGCTTGG-5´	390 bp	56°C
TNFα	5´-CGTCAGCCGATTTGCTATCT-3´<br>3´-CGGACTCCGCAAAGTCTAAG-5´	206 bp	60°C

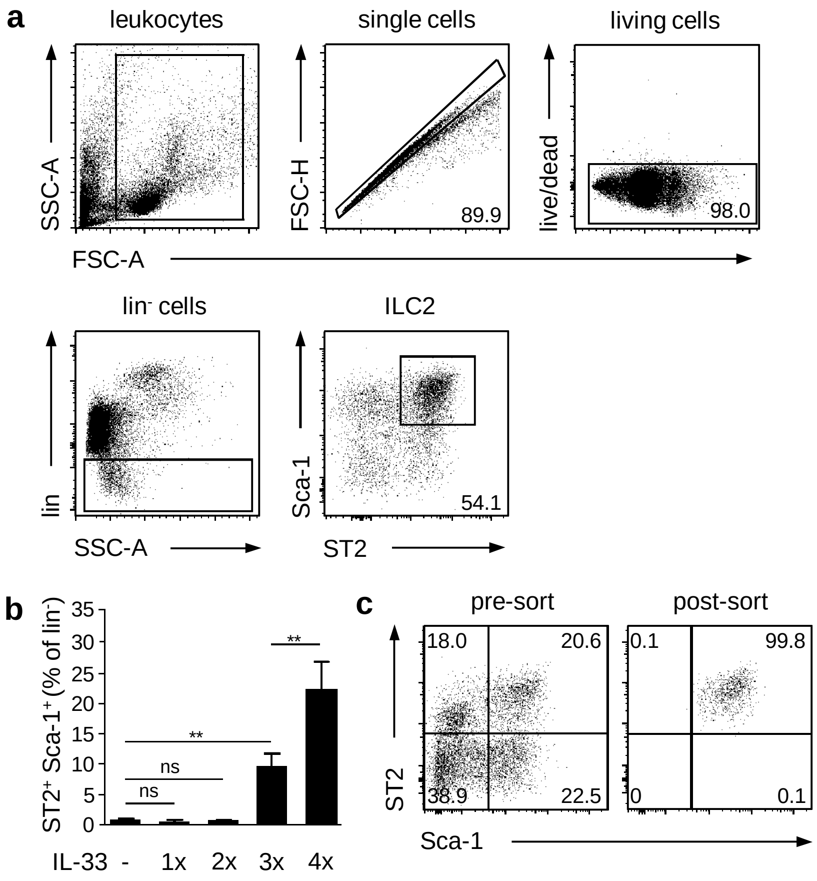

Supplemental Fig. 1. Gating, treatment and sort strategies used for hepatic ILC2 analysis, expansion and isolation. (a) C57BL/6 mice were treated with rmIL-33 on four consecutive days. Living hepatic leukocytes were stained for lin^- $Sca\text{-}1^+$ $ST2^+$ cells to identify ILC2 in liver tissue. (b) C57BL/6 mice were treated with rmIL-33 once a day on up to four consecutive days. Frequencies of hepatic ILC2 were determined by flow cytometry. (c) C57BL/6 mice were treated with rmIL-33 on four consecutive days. Hepatic ILC2 were purely isolated by FACS. Representative dot plots of at least 10 independent experiments are shown. Mean ± SEM of one experiment with four mice per group are shown. One-way ANOVA with post analysis by Tukey-Kramer test. **p< 0.01; ns: not significant

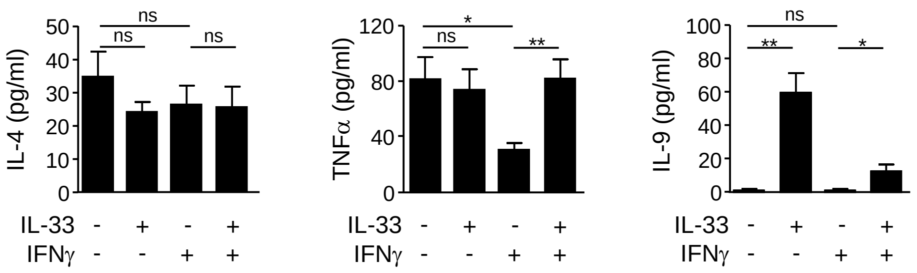

Supplemental Fig. 2. Hepatic ILC2 cytokine expression in response to IL-33 and/or IFNγ. FACS-isolated hepatic ILC2 from IL-33-treated mice were cultured in the presence of IL-33 and/or IFNγ for four days. Cytokine levels were determined in culture supernatants by multiplex assay. Mean ± SEM of 4 independent experiments are shown. One-way ANOVA with post analysis by Tukey-Kramer test. *p< 0.05; **p< 0.01; ns, not significant

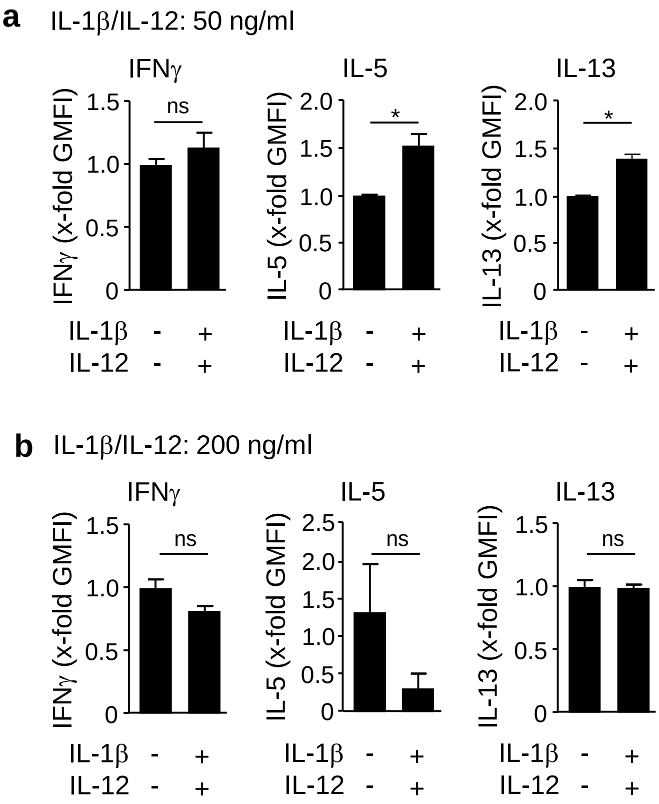

Supplemental Fig. 3. Phenotype of hepatic ILC2 in response to IL-1β/IL-12. Hepatic ILC2 from IL-33-treated mice were cultured in the presence of (a) 50 ng/ml or (b) 200 ng/ml IL-1β/IL-12 for 4 days. Cultures were done in the presence of IL-2 and IL-7. Mean ± SEM of one experiment out of 1-2 experiments are shown. Mann-Whitney U test. *p<0.05; ns, not significant.

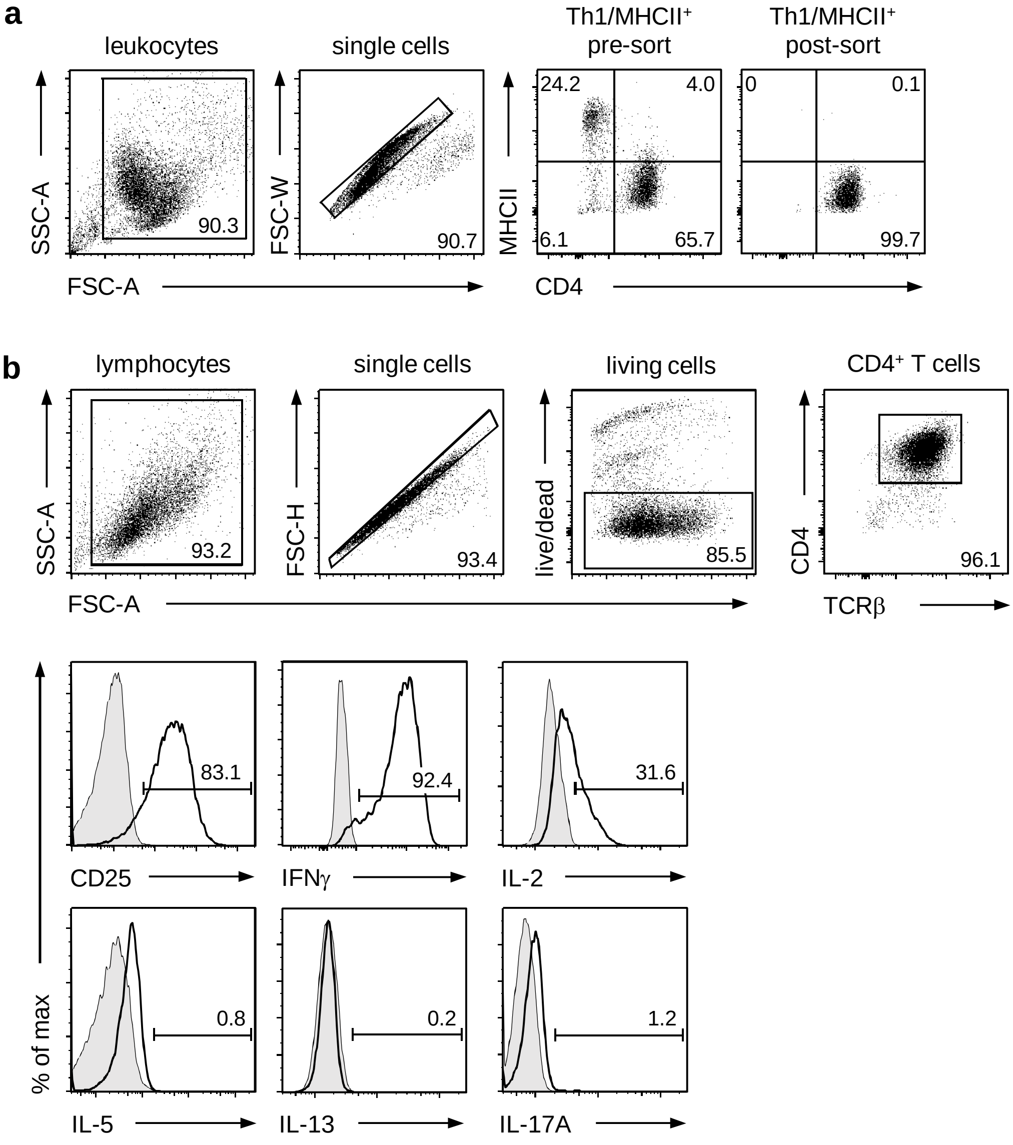

Supplemental Fig. 4. Sort strategy and phenotype analysis of *in-vitro* polarized Th1 cells. OVA-specific $CD4^+$ T cells were co-cultured with splenic $MHCII^+$ cells in the presence of IL-2, IL-12, and OVA for 4 days. (a) Cells were stained for CD4 and MHCII and $MHCII^-$ $CD4^+$ T cells were purely isolated by FACS. (b) $CD4^+$ T cells were stained for CD25, IFNγ, IL-2, IL-5, IL-13, and IL-17A. Histograms show frequencies of activated, cytokine-expressing $CD4^+$ T cells. Bold line, antibody staining; filled graph, fluorescence minus one control Representative histograms and dot plots of 2-3 independent experiments are shown.
